# Upcycling Fishing Net Waste and Metal Oxide from Electroplating Waste into Alga Cultivation Structures with Antibacterial Properties

**DOI:** 10.3390/polym16233415

**Published:** 2024-12-04

**Authors:** Daniel Barros, Luís Nobre, Joana Antunes, João Bessa, Fernando Cunha, Carlos Mota, Fernanda Gomes, Mariana Henriques, Raul Fangueiro

**Affiliations:** 1 Fibrenamics Association, University of Minho, 4800-058 Guimarães, Portugal; luisnobre@fibrenamics.com (L.N.); joaobessa@fibrenamics.com (J.B.); fernandocunha@det.uminho.pt (F.C.); rfangueiro@dem.uminho.pt (R.F.); 2 Beyond Composite, 4410-309 Canelas, Portugal; carlosmota@beyondcomposite.pt; 3 Centre of Biological Engineering, Laboratório de Investigação em Biofilmes Rosário Oliveira, University of Minho, 4710-057 Braga, Portugal; fernandaisabel@ceb.uminho.pt (F.G.); mcrh@deb.uminho.pt (M.H.); 4 LABBELS—Associate Laboratory, 4800-122 Braga, Portugal; 5 Department of Textile Engineering, University of Minho, 4800-058 Guimarães, Portugal

**Keywords:** recycling, antibacterial activity, electroplating waste, sustainability, algae, eco-composite

## Abstract

Plastic waste, especially discarded fishing nets, and electroplating sludges pose significant environmental challenges, impacting marine ecosystems and contributing to pollution. In alga cultivation, invasive microorganisms often hinder growth, necessitating strategies to combat these issues. This study aimed to develop recycled substrates for alga cultivation by repurposing fishing nets and enhancing their surfaces with antibacterial properties using copper oxide (CuO). Additionally, it explores the reuse of CuO from electroplating sludge, providing a sustainable solution that addresses both marine and industrial waste while supporting healthy alga development. Recycled substrates were produced, with different proportions of pure CuO and sludge (1 and 2 wt%) incorporated on the surface. These compositions were processed by hot compression molding and then the antibacterial activity was characterized using a qualitative and quantitative method. The results indicate the possibility of recycling fishing net into new substrates to alga cultivation and the functionalization of their surface using CuO as an antibacterial agent. The antibacterial tests showed a better activity for pure CuO compared to the residual sludge, and better for the higher surface concentration of 2 wt%. Despite the limited bacterial inhibition observed, there is an opportunity for reusing these sludges, typically disposed of in landfills, to obtain specific antibacterial agents that can be applied to the surface of substrates for algal growth.

## 1. Introduction

Plastic waste generated by modern mass consumption practices is a significant contributor to marine pollution, affecting ocean ecosystems on a global scale. This waste comes from various sources, including household trash, fishing activities, discarded ropes, and other plastic materials. Once in the ocean, plastics pose a severe threat to marine life and ecosystems, disrupting their balance and causing widespread damage [1,2,3].

Among the various sources of marine plastic pollution, discarded fishing nets, often referred to as “ghost nets”, are particularly problematic. These nets, abandoned at the end of their useful life, not only pollute the marine environment but also entrap and harm marine species, leading to biodiversity loss [4,5]. The recycling and proper waste treatment of these nets represent a viable approach to reducing their environmental impact and preventing further damage to marine ecosystems [6,7,8]. An innovative strategy involves repurposing these waste materials to create recycled substrates for algae cultivation, providing a dual benefit of waste reduction and resource reuse.

Algae are highly diverse aquatic organisms that inhabit a variety of environments, including oceans, rivers, and lakes, around the world [9]. In natural settings, algae attach to substrates such as rocks, corals, and even artificial surfaces like docks or ropes. This adaptability makes alga cultivation feasible in coastal and offshore areas, although these environments often present significant challenges. Successful alga cultivation depends on factors such as adequate water quality, sufficient light, and nutrient availability, as well as the use of suitable substrates to support alga growth [10,11]. While ropes are traditionally employed as substrates for alga production, there is potential to develop alternative materials that improve seeding, cultivation, and harvesting efficiency.

The cultivation of alga offers numerous environmental, economic, and social benefits. Algae play a vital role in maintaining aquatic ecosystems by contributing to the biological balance and producing oxygen through photosynthesis. Additionally, algae are rich in proteins, minerals, and vitamins, making them valuable for nutritional, medicinal, and cosmetic applications. They can also be used to generate organic matter and fertilizers, contributing to sustainable agricultural practices [9,10,11].

Despite these advantages, alga cultivation faces challenges, particularly due to external factors that can hinder growth and reduce production. Microorganisms such as parasites, bacteria, fungi, and viruses can compete with algae for resources, inhibit their growth, and even cause diseases that lead to cell damage or death [12,13]. For example, *Alphaproteobacteria*, *Gammaproteobacteria*, *Amoebophrya*, and *Labyrinthula* are known to cause cellular damage to algae, while diatoms primarily compete for space and nutrients, further exacerbating cultivation difficulties [14,15,16,17,18]. Addressing these challenges requires exploring innovative methods to mitigate the impact of harmful organisms and enhance alga productivity.

One promising solution is the application of antibacterial finishes to substrate surfaces. These finishes can prevent bacteria and other harmful microorganisms from adhering to the substrates, thereby creating a more favorable environment for alga growth. Such approaches are already widely used in hospital settings, where metallic coatings are employed to reduce the spread of infections by eliminating harmful bacteria and viruses upon contact.

Metals like copper, silver, and zinc, along with their oxides, have demonstrated effective antimicrobial properties and are commonly used in hygienic applications [19]. For instance, copper oxide (CuO) is particularly effective in maintaining its antimicrobial properties even when oxidized. In this project, copper oxide was chosen as the antimicrobial agent for functionalizing recycled substrates used in alga cultivation [20,21]. This CuO is sourced from the reuse of electroplating sludge, an innovative approach that not only addresses alga cultivation challenges, but also contributes to waste management and sustainability.

Electroplating is a vital industrial process used to coat metal parts, but it generates significant waste, particularly in the form of sludge containing heavy metals like copper (Cu), nickel (Ni), zinc (Zn), and chromium (Cr) [22,23]. The improper disposal of this waste can lead to severe environmental contamination, affecting water and soil quality [24,25,26]. Therefore, developing effective methods for treating and reusing this sludge is crucial for mitigating its environmental impact.

This study aimed to develop innovative substrates for alga cultivation using recycled fishing net waste as the base material. To enhance these substrates, their surfaces were modified with antibacterial agents, such as metal oxides, to inhibit the growth of harmful microorganisms. Additionally, the research explores the potential for reusing metal oxides, specifically those extracted from electroplating sludge, as a sustainable source of antibacterial agents. By combining the recycling of ocean plastics with the valorization of industrial waste, this approach not only mitigates environmental pollution, but also contributes to the advancement of sustainable materials for alga cultivation.

## 2. Experimental Procedures

### 2.1. Materials

To produce the substrates for alga cultivation, waste fishing nets were acquired from the entity Peniche Ocean Watch, Portugal. To produce the substrates with antimicrobial properties, pure CuO microparticles were used and acquired from Sigma-Aldrich, Lyon, France, along with residual sludge containing about 10 wt% of copper oxide, originating from the electroplating process, supplied by an entity located in the Braga district. These materials allowed for producing antibacterial substrates to develop alga cultivation substrates.

### 2.2. Sample Development

Different combinations of recycled fishing nets functionalized with CuO were produced via the compression molding process, using fishing net waste, pure CuO and residual sludges from the electroplating processes. The samples were developed by adding 1 and 2 wt% of CuO/sludge, that was added on the recycled substrates previously, produced by hot-pressing. The samples were produced at 220 to 230 °C (melting temperatures of polyamide, composition material of the fishing nets), and the functionalized coatings were added applying it into the mold surface before the hot-pressing process.

**Figure 1:**
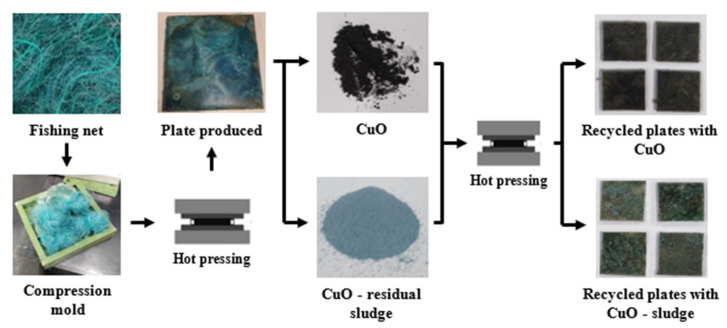
Schematic representation of the sample production.

In Figure 1, a schematic representation of the production cycle for different sample combinations is presented.

The addition of antibacterial elements was performed on each plate surface in 1:99 and 2:98 weight percentage (1% and 2%, respectively), as listed in Table 1. Then, each combination of plates was cut in 2 × 2 cm specimens to perform antibacterial analysis.

### 2.3. Antibacterial Activity

The antibacterial activity test was carried out using two methods, a qualitative test and a quantitative test based on international standards: modified JIS L 1902:2008 standard (Testing for antibacterial activity and efficacy on textile products) [27] and ISO 22196 standard (Plastics—Measurement of antibacterial activity on plastic surfaces) [28], respectively. Samples measuring 2 × 2 cm of various compositions were considered for testing, with reference samples comprising substrate without antimicrobials and the different antibacterial agents used (powder). All samples were sterilized by ultraviolet radiation for 30 min on each side.

Both of the testing methods used a Gram-positive and a Gram-negative bacterial strain: *Staphylococcus aureus* ATCC 6538 and *Escherichia coli* ATCC 25922, respectively. This analytical method was selected to evaluate the antibacterial activity of the additive agents applied to the surface during the substrate production process. According to the literature, these two bacteria are commonly used to assess antibacterial capacity.

The halo test, also known as the disk diffusion test, was performed as follows. An inoculum was incubated in a tryptic soy broth (TSB) overnight at 37 °C and 120 rpm. Then, 1 mL from the inoculum with 1 × 10^6^ cells/mL was added to 15 mL of tryptic soy agar (TSA) warmed at 45 °C. This solution was disposed in a sterilized Petri dish. After medium solidification, the samples were placed over the agar and incubated for 24 h at 37 °C. For powdered antimicrobials, a 10 mg sample was used. The evaluation of the antibacterial activity was made based on the observation of the zone of inhibition, around and under the samples.

The quantitative test was performed as follows. With 1 × 10^6^ cells/mL, 200 μL of inoculum was placed onto the surfaces of the coated reference samples. The bacterial inoculum was covered with a plastic cover film (1 × 1 cm). The samples were incubated for 24 h at 37 °C. After this period of incubation, the cells were collected by adding 10 mL of a washing solution (TSB + 7.0 g/L of Tween 80). Serial dilutions were made and then plated on TSA for 24 h at 37 °C. The number of colony-forming units (CFUs) was determined.

Antibacterial activity (R) was determined by calculating according to Equation (1):

R = Ut − At (1)

where “Ut” is the average of the logarithm of the number of viable bacteria (N), in cells/cm^2^, taken from the control samples after 24 h and “At” is the average of the logarithm of the number of viable bacteria (N) in cells/cm^2^, taken from the functionalized samples after 24 h. Qualitative and quantitative analysis comprised three independent assays performed in duplicate.

### 2.4. Mechanical Characterization

The mechanical characterizations carried out were tensile and flexural tests, performed in an universal test machine (Hounsfield H100KS). For these tests, a 2.5 kN load cell and a crosshead speed of 2 mm/min were used in accordance with the ISO 527-4 (Plastics—Determination of tensile properties) [29] standard for tensile tests and the ISO 178 (Plastics—Determination of flexural properties) standard for flexure test [30].

### 2.5. Fourier Transform Infrared Spectroscopy (FTIR) Characterization 

The chemical composition of the sample production was analyzed using Fourier transform infrared spectroscopy (FTIR) coupled with the attenuated reflection (ATR) technique using SHIMADZU—IRAffinity-1S, Kyoto, Japan. All the spectra were obtained in the transmittance mode with 45 scans over a wavenumber range of 4000–400 cm^−1^. This technique will allow for comparing the chemical compositions of the produced fishing net recycled substrates with and without antibacterial agent.

### 2.6. Scanning Electron Microscopy (SEM) and Elemental Analyses

The specimen’s surface morphology evaluation was made using the Scanning Electron Microscopy (SEM); each is a technique widely used in scientific research to visualize the morphology of materials at the micro- and nanoscales. It employs an electron beam to generate high-resolution images of the sample surface. In this study, SEM analysis was conducted into specific area of different specimens using an SEM instrument, FEI Nova, 200, Hillsboro, OR, USA, to evaluate the surface differences of the fishing net recycled surface with and without antibacterial agent. Additionally, by performing an elemental analysis of the developed samples and the antibacterial agents used, it is possible to evaluate their composition and determine the elements they are made of. These elemental analyses were made using an EDS instrument—Pegasus X4M (EDS/EBSD).

### 2.7. Fourier Transform Infrared Spectroscopy (XRD) Characterization

X-ray diffraction (XRD) is a powerful analytical technique used to investigate the crystalline structure of materials by examining the diffraction pattern produced when X-rays interact with the sample’s atomic planes. This technique was conducted using an XRD instrument—Bruker D8 Discover. By analyzing the diffraction patterns, it was possible to assess the structural integrity and determine the crystalline phases present in each sample, providing insights into the effectiveness and purity of CuO obtained from different sources.

### 2.8. Surface Roughness Characterizaiton

Surface roughness testing was performed on the recycled fishing net substrates, in accordance with ISO 21920-2 standards [31]. This testing utilized a Mitutoyo SJ-210, Kanagawa, Japan, which allowed for the measurement of the arithmetic average roughness (Ra), a parameter that represents the average surface profile deviations from the mean line. This evaluation enabled a comparison of the effects of different antibacterial agents on surface roughness.

### 2.9. Water Absorption Characterizaiton

Water absorption tests were conducted on samples of recycled fishing nets with and without antibacterial agents, aiming to evaluate the impact of these agents on the water absorption properties of the recycled substrates. The test was conducted following the ASTM D570 standard [32], where the samples were fully immersed in distilled water for a 24 h, and the percentage of water absorbed was calculated according to Equation (2):

(2) $$\mathrm{Water}\;\mathrm{absorption}\;(\%)=\frac{W_{d}-W_{w}}{W_{w}}\times 100$$

where

W_d_ = dry mass of the sample;
W_w_ = wet mass of the sample.

## 3. Results and Discussion

### 3.1. Measurement of Antibacterial Activity on Recycled Fishing Net Substrate Surfaces

The antibacterial activity of recycled fishing nets functionalized with pure CuO and sludge was evaluated against standard strains, including the Gram-positive bacteria *Staphylococcus aureus* and the Gram-negative bacteria *Escherichia coli*.

**Figure 2:**
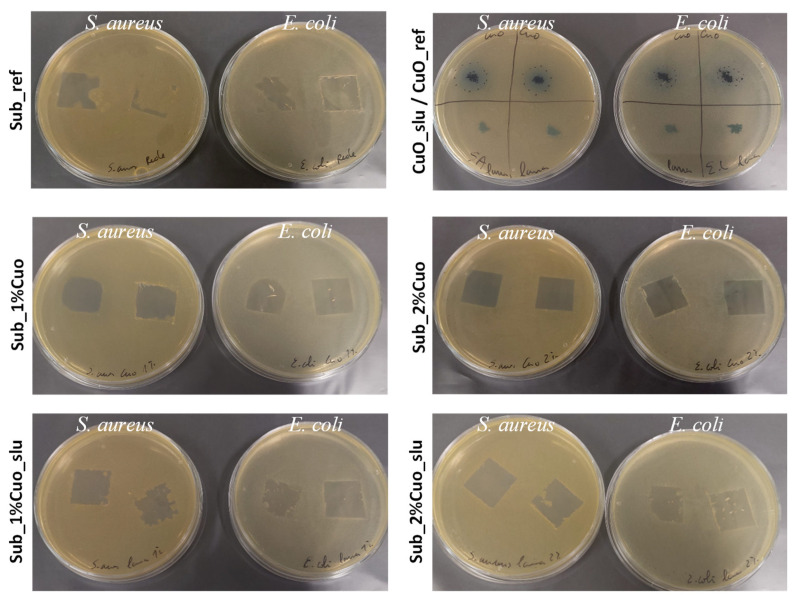
Qualitative halo test results for reference and coating samples, and antibacterial powders.

In Figure 2, the results of the antibacterial activity are presented according to the qualitative method, where visually and through the halo test, it was possible to assess the substrate’s ability to inhibit bacterial growth, providing a comprehensive understanding of the antibacterial effectiveness of each sample.

When considering specifically the recycled network substrate (Sub_ref), a significant absence of antibacterial activity against the tested bacterial strains was observed. The lack of antibacterial efficacy may be attributed to the nature of the recycled material, which showed to not possess intrinsic properties capable of inhibiting bacterial growth. Also, for the CuO_slu_ref sample, no halo formation was visible.

In contrast, the pure antibacterial agent CuO (CuO_ref) demonstrated robust antibacterial activity, evidenced by the formation of clear halos around the areas of contact with the bacteria (*S. aureus*: 16 mm and *E. coli:* 11 mm), indicating an effective inhibition of bacterial growth.

When these antibacterial agents were added to the surface of the recycled network substrate at 1 and 2 wt% concentrations, it was found that substrates previously without any or low antibacterial activity exhibited some activity. Comparing the two concentrations considered, a higher activity was observed for the 2 wt% concentration, as it contains a higher concentration of the antibacterial agent, leading to better efficacy in eliminating bacteria. From the results of the qualitative assays, a greater antibacterial efficacy was observed for the CuO_ref and sub_2% CuO samples.

The results obtained using the quantitative method, which determined the antibacterial activity (R), are presented in Table 2.

Based on the results from Table 1, and although R < 2 (the minimum threshold to consider antibacterial activity occurs), it was observed that substrates functionalized with 2 wt% pure CuO exhibited indeciduous of some bacterial inhibition, whereas non-functionalized and CuO_Slu substrates showed no activity (R = 0). With the increase in antibacterial agent concentration on the surface from 1 to 2 wt%, an increase in the R was observed, especially for the samples functionalized with pure CuO. This is attributed to a greater exposure of these agents to bacteria, thereby enhancing their effectiveness.

In substrates coated with pure CuO, higher bacterial inhibition was noted against *S. aureus*. Substrates with CuO from residual sludge exhibited no bacterial inhibition against *S. aureus* and *E. coli*. This must be attributed to the difference in CuO concentration in each functionalization, as pure CuO substrates have a concentration of 100%, while residual sludge substrates have approximately 10 wt% copper oxide concentration (according to the supplier). Accordingly, when comparing bacterial inhibition among the residues, better results were observed for samples with pure CuO. As for samples functionalized with residual sludge, despite exhibiting lower antibacterial activity, the results obtained may be considered as positive, giving that it is a residue from galvanoplasty processes, where copper oxide present at concentrations of approximately 10 wt%.

These results may indicate the feasibility of reusing residual sludge from galvanoplasty processes, thereby avoiding their disposal in landfills. Additionally, it is suggested that using higher concentrations of CuO may increase the R value, thereby enhancing the antibacterial capacity of the surface, or test the antibacterial activity by testing other sludges from different processes that may contain other elements capable of possessing antibacterial properties.

### 3.2. Mechanical Properties

**Figure 3:**
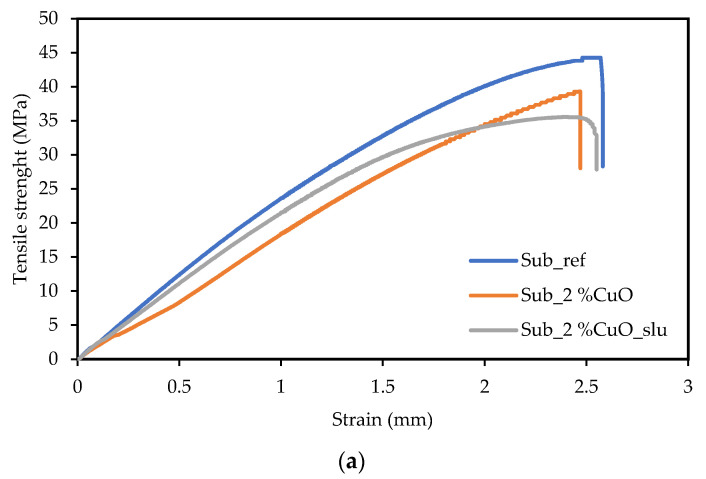

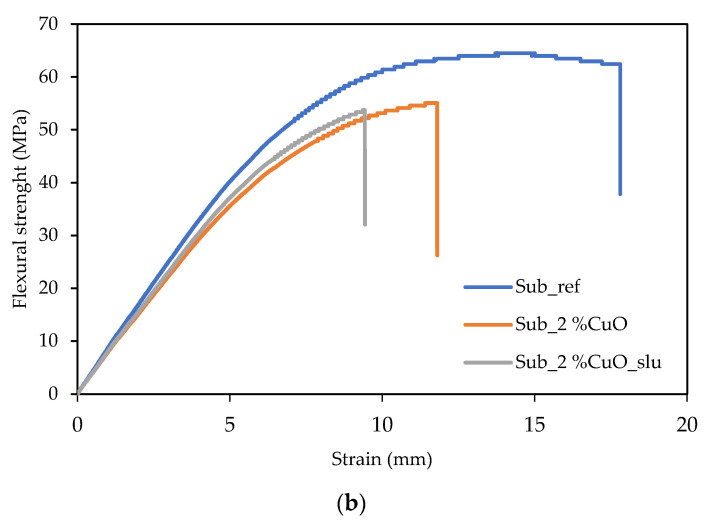
Mechanical characterization results: (**a**) tensile and (**b**) flexural.

For the mechanical characterization tests, tensile and flexural tests were conducted. Specimens were cut into bone and rectangular shapes according to the respective standards. The results are presented in Figure 3.

According to the tensile results, it is possible to verify a small decrease in tensile strength when adding 2% of antibacterial agents, 11.3% to the addition of 2% CuO, and 19.6% to the 2% CuO from sludge. Like the findings of the tensile tests, there was a small decrease of 14.6% in flexural strength for the samples with an addition of 2% CuO and 16.6% to the 2% CuO from residual sludges.

For both additions of 2% antibacterial agent, a slight decrease in tensile and flexural strength was observed. This may be related to the addition of these agents to the surface of recycled fishing net substrates. During the surface functionalization process, these substrates undergo thermal reprocessing, which can lead to a loss of properties in the polymeric matrix. The samples with 2% CuO derived from electroplating sludge residues exhibited lower resistance compared to the samples with pure CuO, due to the presence of impurities.

### 3.3. Fourier Transform Infrared Spectroscopy (FTIR)

**Figure 4:**
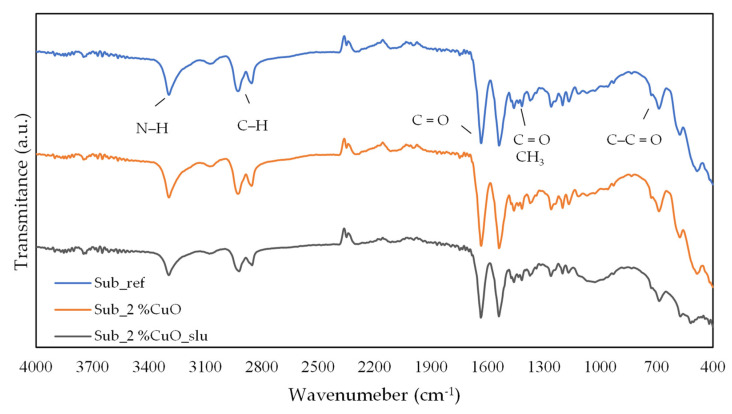
FTIR spectra of the fishing net substrates with and without antibacterial agent.

In this section its presented FTIR test results for the different specimens produced to evaluate the influence of adding 2 wt% of antibacterial agent in the surface. The results are presented in Figure 4.

The FTIR spectrum of the polyamide shows characteristic bands:

- Around 3300 cm^−1^: stretching vibration of the amine groups (at ca. 3075 cm^−1^, the overtone of N–H bending has a signal [33];
- Around 2925 and 2854 cm^−1^ (aliphatic C–H stretching), 1636 cm^−1^ (C=O stretching), and 1475 cm^−1^ (aromatic C=C), and 1405 cm^−1^ (C–N stretching);
- Around 1634 cm^−1^ and 1535 cm^−1^ presents the (C=O) and (N–H) bands;
- peaks appearing at 1474 cm^−1^ and 1370 cm^−1^: C-H asymmetric bending in (–CH_2_) or (–CH_3_–) and symmetric bending in (–CH_3_–), respectively;
- At about 834 cm^−1^ and 686 cm^−1^: C-C=O stretching vibration and N–H out-of-plane bending vibration, respectively [34];

After analyzing the FTIR results and comparing them with the spectra available in databases, it is confirmed that the obtained spectrum corresponds to that of polyamide (PA) [35].

Comparing the spectra of recycled substrates with those functionalized with antibacterial agents (both pure and residual), no significant differences were observed in the obtained spectra. The primary distinction lies in the peak intensity, which decreased when 2 wt% of the antibacterial agent was added, as its presence “hinders” signal detection, leading to reduced peak intensity.

### 3.4. Scanning Electron Microscopy SEM and Elemental Analyses

**Figure 5:**
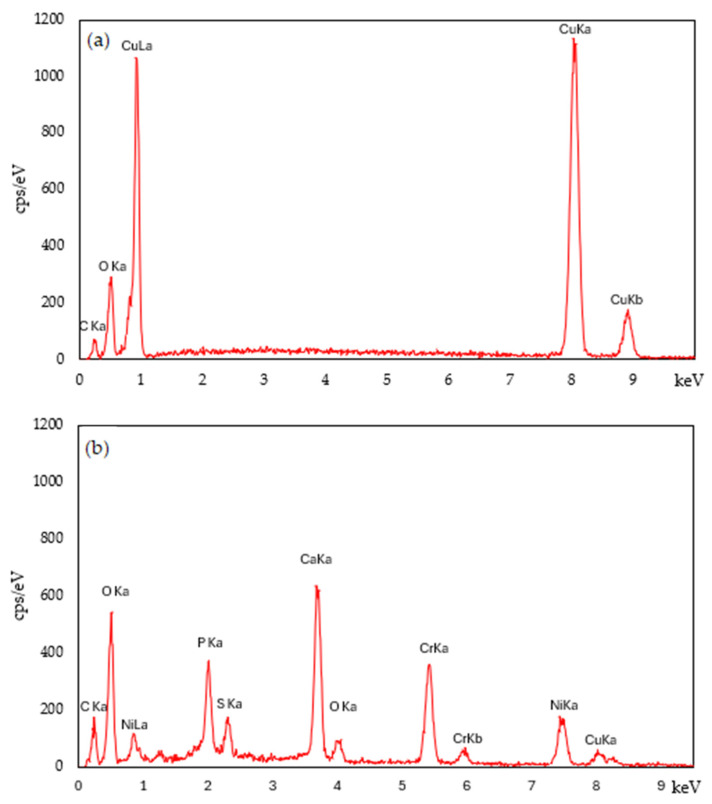
EDS spectra of each antibacterial agent: (**a**) pure CuO and (**b**) sludge from the electroplating process.

Using SEM/EDS analysis, it was possible to evaluate the surface of the samples with and without the addition of antioxidant agent, to assess the dispersion of these agents on the surface and evaluate the present elements in each surface composition. Firstly, is presented the results of the elemental analysis performed by EDS, exclusively for the antibacterial agents, especially to understand the composition of the electroplating residue, as presented in Figure 5, and the chemical composition in wt% of each element in Table 3.

The EDS analysis confirmed that pure CuO is primarily composed of copper (Cu) at energy levels of 0.9, 8.1, and 8.9 keV, and oxygen (O) at 0.6 keV. From the obtained results, it is evident that the sludge resulting from the electroplating process contains a wide array of elements identified at various peaks: carbon (C) at 0.2 keV, oxygen (O) at 0.6 keV, nickel (Ni) at 0.9 and 7.5 keV, magnesium (Mg) at 1.3 keV, phosphorus (P) at 2 keV, sulfur (S) at 2.3 keV, calcium (Ca) at 3.7 and 4 keV, chromium (Cr) at 5.4 and 6 keV, and copper (Cu) at 8 and 9 keV.

Through the chemical analysis, the mass percentage of each element detected in both antibacterial agents was determined. In the CuO powders, a pronounced presence of Cu and O was observed, as expected due to the use of pure powder. In the electroplating sludge powder, several elements were detected, with a notable presence of Ca, Cr, and Ni, which appear in the composition as oxides, justified by the high oxygen content.

**Figure 6:**
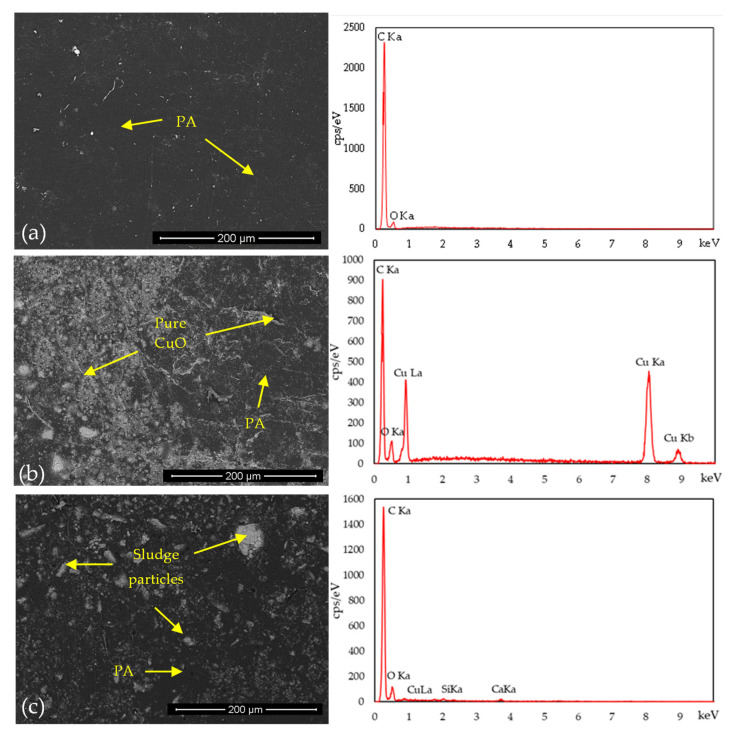
SEM images and EDS spectrs of the fishing net substrates: (**a**) reference, (**b**) with 2% CuO and (**c**) with 2%CuO_slu.

SEM results of recycled fishing net substrates, with and without 2 wt% antibacterial agents, are presented in Figure 6, along with the corresponding EDS analysis to better understand the two antibacterial agents.

The morphological and elemental characterization of the reference substrate, produced exclusively from fishing nets (Figure 6a), reveals a smooth and homogeneous surface composed of polyamide (PA), the primary constituent of the nets. A few white spots are visible on the surface, likely representing residual contaminants from the fishing nets, which remained despite the washing and grinding processes. These residues persisted through compression molding and became embedded within the substrate, contributing to its final surface characteristics. Through EDS, it is possible to identify the elements carbon (C) (0.26 keV) and oxygen (O) (0.56 keV), which are characteristic of the chemical structure of polyamide.

The SEM results to the substrate surface with the addition of 2 wt% pure CuO (Figure 6b), a good distribution of particles across the surface is observed, though it is not entirely homogeneous. Certain areas exhibit a higher density of pure CuO particles, with small clusters also present. EDS analysis further confirms the presence of carbon (C) and oxygen (O) peaks, while the addition of pure CuO is evident from the appearance of copper (Cu) peaks at 0.9, 8.1, and 9 keV.

From the SEM images of the specimens with 2% CuO_slu (Figure 6c), it was also possible to observe a good distribution of particles. However, the presence of agglomerates suggests that the addition process of these agents to the surface needs optimization to achieve a more homogeneous antibacterial surface. Based on the EDS results, the presence of various elements detected in the analysis of the residual sludge alone can be confirmed. However, the identified peaks display much lower energy levels. This indicates that these elements are present on the substrate surface, but their lower concentration resulted in reduced antibacterial activity of these substrates, due to the weak presence of CuO.

SEM images further show a greater presence of CuO compared to CuO_slu on the substrate surfaces, due to the better distribution and adhesion of CuO microparticles compared to the larger CuO_slu macroparticles.

### 3.5. Fourier Transform Infrared Spectroscopy (XRD)

**Figure 7:**
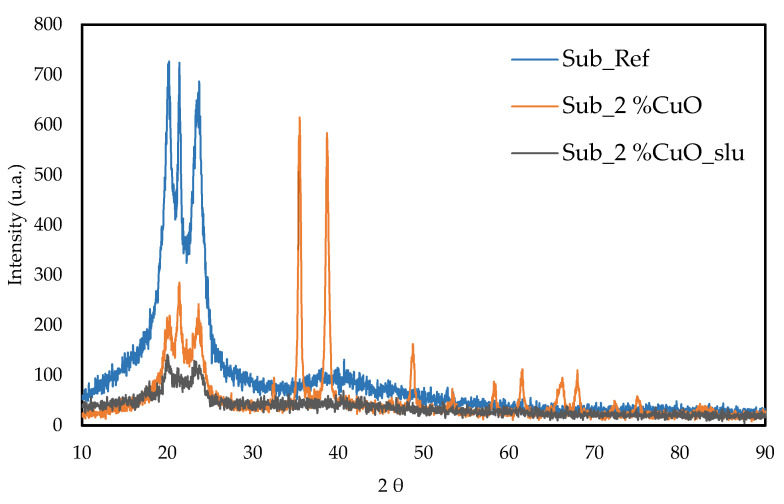
X-ray diffraction (XRD) of the reference fishing nets substrates, with 2% CuO and 2%CuO_slu.

The phase composition and element determination were analyzed by the XRD pattern, shown in Figure 7.

The sample Sub_Ref was analyzed by X-ray diffraction (XRD) to determine its composition and crystalline structure. The results show two main peaks at 2θ = 20.4° and 2θ = 23.6°, corresponding to the (200) and (002) crystallographic planes, confirming the presence of polyamide. The absence of additional peaks suggests a typical semicrystalline structure with high purity and no significant crystalline phases. These crystallographic planes indicate a characteristic polyamide organization, with well-defined crystalline regions, confirming the material’s identity (X) [36,37].

The sample 2 %CuO was analyzed by X-ray diffraction (XRD) to determine its composition and crystalline structure. The results show two main peaks at 2θ = 35.3° and 2θ = 38.6°, corresponding to the crystallographic planes (111) and (022), indicating the presence of CuO. Additional less intense peaks were observed at 2θ = 48.84°, 53.5°, 58.4°, 61.6°, 66.2°, and 68°, corresponding to the crystallographic planes (202), (202), (113), (022), (220), and (222), according to the literature [38,39]. Furthermore, peaks corresponding to polyamide were also detected, resulting from readings over the substrate on which CuO was deposited during sample processing, confirming the presence of polyamide on the material’s surface.

For the sample Sub_2% CuO_slu, only the characteristic peaks of polyamide were observed, while the other elements detected in the EDS analysis did not appear in the spectrum, which displayed an increase in noise. This may indicate that there is no significant crystalline material present in the analyzed plating sludge, or that if it exists, its concentration is too low, rendering the peaks undetectable. Additionally, as this is a waste material, the surface of the substrate may be contaminated, complicating peak visualization. The degradation of the material or alterations in the crystalline structure, resulting from chemical or thermal processes, may also contribute to the absence of peaks in the spectrum.

### 3.6. Surface Roughness

**Figure 8:**
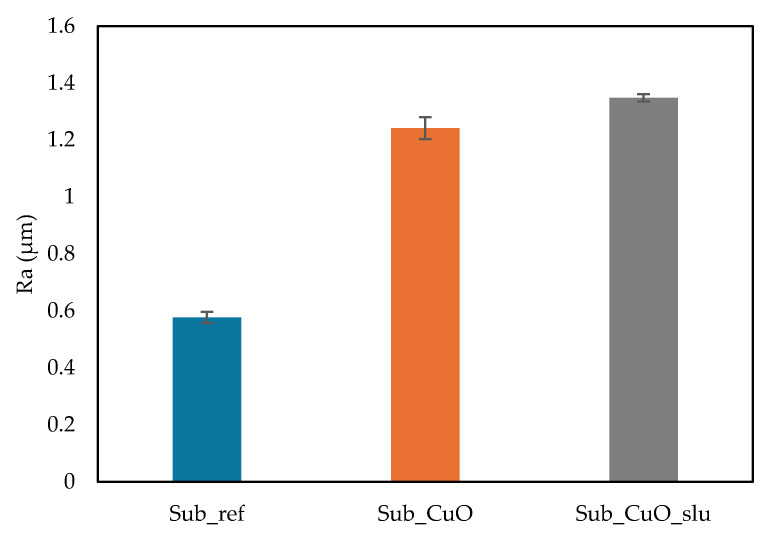
Surface arithmetic average roughness (Ra) of the reference fishing net substrates, with 2% CuO and 2%CuO_slu.

In this section, the surface roughness test results for the different specimens produced to evaluate the influence of adding 2 wt% of antibacterial agent in the surface are presented, as shown in Figure 8.

The results reveal a significant increase in the roughness of the additive surfaces compared to the reference surface made from fishing net waste. The surface of the Sub_ref sample exhibited an arithmetic average roughness (Ra) value of 0.578 µm, while the Sub_2%CuO sample, which includes 2% by mass of pure CuO microparticles, reached 1.242 µm. The Sub_2%CuO_slu sample, containing 2% by mass of macroparticles derived from electroplating process waste—which includes various oxides, as verified by EDS and XRD—showed an Ra of 1.349 µm.

The comparison between the additive samples indicates that the addition of macroparticles results in an even greater increase in roughness compared to the addition of CuO microparticles. This increased roughness is advantageous for algal cultivation, as rougher surfaces enhance algal adhesion, thereby promoting their growth in aquatic environments.

### 3.7. Water Absorption

**Figure 9:**
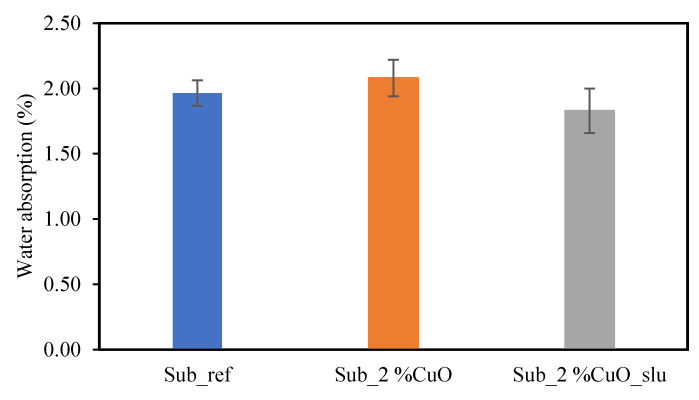
Water absorption results.

For the water absorption tests, the samples were weighed when dry and after being submerged in water to calculate the percentage of water absorption retained. The obtained values are presented in Figure 9.

After the water absorption tests, no differences were observed between the substrates made with fishing nets, regardless of whether 2 wt% of the antibacterial agent was applied. The percentage of water absorption obtained was approximately 2%. The standard deviations were around 0.14, indicating high reproducibility in the tests performed, as well as in the samples produced. The addition of the antibacterial agent to the surface of the substrates made from fishing net residue did not affect water absorption in any way.

## 4. Conclusions

In summary, the results highlight that the surface functionalization treatment of substrates, derived from recycled fishing nets, with antibacterial agents yielded several significant findings:

- Enhanced antibacterial capacity observed in the surface-treated substrates.
- Despite the obtained values being R > 2 for the minimum threshold to be considered antibacterial activity, a greater potential of bacterial elimination efficiency exhibited by pure CuO compared to residual sludges from electroplating processes was observed.
- Surface treatment with pure CuO 2 wt% demonstrated superior capacity of possible bacterial inhibition compared to 1 wt% due to the obtained results.
- Despite the low levels of antibacterial activity, by adjusting the concentrations of the antibacterial agent, the results obtained show promising indicators for the potential reuse of residual sludges in developing solutions with antibacterial capabilities.
- A slight decrease in mechanical strength was observed in samples with 2% pure CuO and 2% CuO from electroplating sludge (CuO_slu). This reduction is due to the reprocessing of the substrates after surface modification.
- FTIR analysis confirmed that the substrates are made of polyamide (PA). The detected peaks did not change significantly, but their intensity was reduced, indicating that the substrate structure remained unchanged after the addition.
- EDS identified the elements in the antibacterial agents. SEM showed the distribution of these elements on the surface and their morphology.
- XRD confirmed the composition of the substrates. Cu and O were found in the pure CuO sample, while additional elements, including Cu and O, were identified in the sample with electroplating residues.
- Roughness tests showed a significant increase in samples with pure CuO and CuO_slu. This higher roughness benefits alga growth by providing better conditions for adhesion and development.
- Water absorption tests showed no significant differences between samples. This was expected, as the agents were added in low concentrations and only on the surface, without altering substrate porosity.
- This preliminary study allowed us to verify the promising antibacterial capacity of CuO, with the next steps being the evaluation of its antibacterial activity during alga cultivation in a controlled environment.

The results obtained for the antibacterial agents added to the surface of the polymeric substrates demonstrated their potential in eliminating bacteria that may colonize the surface. This validates the feasibility of the deposition method employed during the substrate production process at the laboratory scale. Furthermore, additional characterizations confirmed that the necessary conditions were established to develop substrates suitable for the cultivation and growth of algae. As the next steps, we are considering evaluating larger quantities of the antibacterial agent and conducting these tests using bacteria in real environmental conditions, alongside the assessment and monitoring of alga growth.

## Figures and Tables

**Table 1:** Produced samples.

Sample	Antibacterial Agent	Sample Reference
Recycled fishing net (only)	-	Sub_ref
Antibacterial powder	CuO	CuO_ref
	CuO sludge	CuO_slu_ref
Recycled fishing net	1% CuO 2% CuO 1% CuO sludge 2% CuO sludge	Sub_1%CuO Sub_2%CuO Sub_1%CuO_slu Sub_2%CuO_slu

**Table 2:** Antibacterial activity (R) of functionalized substrates with 1 and 2 wt% of pure CuO and sludge against *Escherichia coli* and *Staphylococcus aureus*.

Functionalization	*E. coli*	*S. aureus*
Sub_ref	0	0
Sub_1%CuO	0	0.7 ± 0.1
Sub_2%CuO	0.7 ± 0.64	1.3 ± 0.55
Sub_1%CuO_slu	0.3 ± 0.02	0.2 ± 0.05
Sub_2%CuO_slu	0.4 ± 0.09	0.3 ± 0.11

**Table 3:** Chemical composition (EDS) of pure CuO and electroplating sludge.

Sample	Element (wt%)									
	C	O	Cu	Mg	P	S	Ca	Cr	Ni	Total
CuO	8.54	13.89	77.57	-	-	-	-	-	-	100
Sludge	16.09	37.99	3.7	0.88	5.47	2.2	11.51	11.57	10.59	100

## Data Availability

All data are contained within this article.
